# Supplementary material for: Prognostic values of GMPS, PR, CD40, and p21 in ovarian cancer
Source: PeerJ. 2019 Jan 25;7:e6301. doi: 10.7717/peerj.6301 (PMC6348951; doi:10.7717/peerj.6301)
Supplement: Supplemental Information 1 [file peerj-07-6301-s001.docx]

Figure1 B1 Colony formation efficiency in soft agar(%)

Raw data

| MOSE-I | MOSE-II |
| --- | --- |
| 0 | 11.031 |
| 0 | 9.896 |
| 0 | 10.665 |

|  | Mean | SD | n | **t** | ***P*** |
| --- | --- | --- | --- | --- | --- |
| MOSE-I | 0 | 0 | 3 | >9999 | <0.0001 |
| MOSE-II | 10.53 | 0.58 | 3 |  |  |

Figure1 C1 Colony formation efficiency (%)

Raw data

| MOSE-I | MOSE-II |
| --- | --- |
|  |  |
| 3.985 | 12.219 |
| 5.107 | 11.375 |
| 4.325 | 10.173 |

|  | Mean | SD | n | t | *P* |
| --- | --- | --- | --- | --- | --- |
| MOSE-I | 4.47 | 0.503 | 3 | 10.26 | 0.0005 |
| MOSE-II | 11.26 | 1.03 | 3 |  |  |

Figure1 C2 colony formation diameter

Raw data

| MOSE-I | MOSE-II |
| --- | --- |
| 2.461 | 5.435 |
| 0.911 | 6.068 |
| 1.489 | 5.404 |
| 0.869 | 4.679 |
| 0.974 | 5.855 |
| 2.087 | 5.874 |
| 1.255 | 8.145 |
| 1.33 | 6.044 |
| 2.131 | 2.746 |
| 2.748 | 3.278 |
| 2.734 |  |
| 2.942 |  |
| 0.911 |  |
| 0.932 |  |
| 0.735 |  |
| 1.869 |  |
| 1.345 |  |
| 1.019 |  |
| 1.592 |  |

|  | Mean | SD | n | t | *P* |
| --- | --- | --- | --- | --- | --- |
| MOSE-I | 1.597 | 0.74417 | 18 | 8.816 | <0.0001 |
| MOSE-II | 5.35 | 1.522903 | 10 |  |  |

Although both Figure1B1 and C1 data are clone formation rates, the denominators of all their values are the same. So all variables are numerical variables and are shown as Mean±SD. Unpaired-t tests were used to assess differences in MOSE-I and MOSE-II cell lines. *,**or ***= significantly different from two cell lines, P<0.05,P<0.001 and P<0.0001,respectively. All statistical analyses were conducted using SAS software version 9.4 (SAS Institute, Inc., Cary, NC, USA).
